# Closed Versus Open Vent Port Techniques and the Disconnection Technique for Optimizing Lung Collapse With Bronchial Blockers in Video‐Assisted Thoracoscopic Surgery: A Randomized Controlled Trial

**DOI:** 10.1111/crj.70235

**Published:** 2026-10-22

**Authors:** Juan Yao, Ya‐Jing Chen, Fang‐Ming Shen, Jue Xie, Jiao‐Jiao Yang, Lu‐Xin Huang, Xiao‐Xiang Tan, Ying Yang, Jie Sun

**Affiliations:** ^1^ Department of Anesthesiology, Surgery and Pain Management, Zhongda Hospital Southeast University Nanjing Jiangsu China; ^2^ Jiangsu Provincial Key Laboratory of Critical Care Medicine Nanjing Jiangsu China

**Keywords:** bronchial blocker, lung collapse, one‐lung ventilation, video‐assisted thoracoscopic surgery

## Abstract

**Introduction:**

For patients undergoing video‐assisted thoracoscopic surgery (VATS) for lung cancer or other pulmonary lesions, effective one‐lung ventilation (OLV) is essential to provide surgical exposure. Bronchial blockers (BBs) are increasingly used for OLV, but the optimal management of their vent port to accelerate lung collapse remains unclear.

**Methods:**

In this prospective, randomized, controlled trial, 75 patients were allocated to three groups (*n* = 25 each): Group 1—BB with vent port closed; Group 2 (control)—BB with vent port open; Group 3—BB with the disconnection technique. Primary outcome was the time to complete lung collapse, which was scored according to the video clips obtained during the operation. Secondary outcomes included intraoperative hypoxemia, blood gas analysis, blocker malposition, perioperative complications, and surgeon satisfaction.

**Results:**

A total of 72 patients were included in the modified intention‐to‐treat analysis, with 24 patients in each group. Median time to complete lung collapse was 15 min (95% CI: 10–15) in Groups 1 and 3 but was not reached in Group 2 (15/24, 62.5% had not achieved complete collapse by 30 min). In an exploratory 30‐min restricted mean survival time (RMST), complete collapse occurred on average 14.17 min earlier in Group 1 (95% CI: 11.10–17.23) and 12.50 min earlier in Group 3 (95% CI: 9.62–15.38) than in Group 2, with no difference between Groups 1 and 3 (1.67 min, 95% CI: −1.85 to 5.19); time‐averaged hazard ratios were consistent (Group 1 vs. 2, 9.80 [4.22–22.73]; Group 3 vs. 2, 7.63 [3.36–17.31]; Group 1 vs. 3, 1.20 [0.67–2.16]). No significant differences were detected in hypoxemia, blocker malposition, or complications across groups; however, the trial was not powered for safety outcomes.

**Conclusion:**

In patients aged 18–65 years with ASA I–II and normal pulmonary function undergoing thoracic surgery, the closure of the BB vent port results in a more rapid lung collapse compared to maintaining the vent port open. However, there was no statistically significant difference observed between the vent port closure technique and the disconnection technique regarding the time required for complete lung collapse.

**Trial Registration:**

ChiCTR identifier: ChiCTR2000039896

## Graphical abstract

In patients (18–65 years, ASA I–II, normal lung function) undergoing thoracic surgery, closing the BB vent port results in a more rapid lung collapse compared to maintaining the vent port open. However, no statistically significant difference was observed between the vent port closure technique and the disconnection technique regarding the time required for complete lung collapse.

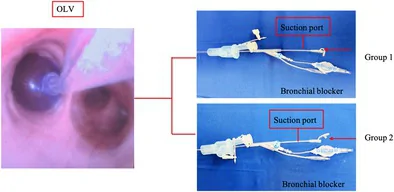

**Abbreviations**
ASA: American Society of Anesthesiology
BB: bronchial blocker
BMI: body mass index
CPAP: continuous positive airway pressure
FEV1/FVC: forced expiratory volume in 1 s/forced vital capacity
FiO_2_
: fraction of inspired oxygen
FOB: fiberoptic bronchoscope
HR: heart rate
IBW: ideal body weight
ICU: intensive care unit
MAP: mean arterial pressure
OLV: one‐lung ventilation
PaCO_2_
: partial pressure of carbon dioxide
PACU: post‐anesthesia care unit
PaO_2_
: partial pressure of oxygen
PEEP: positive end‐expiratory pressure
pH: potential of hydrogen
RMST: restricted mean survival time
SaO_2_
: saturation of oxygen
SLT: single‐lumen endotracheal tube
SpO_2_
: blood oxygen saturation
TLV: two‐lung ventilation
VATS: video‐assisted thoracoscopic surgery

## 1 Introduction

The development of video‐assisted thoracoscopic surgery (VATS) has enabled the use of one‐lung ventilation (OLV) to improve surgical exposure in intrathoracic operations. The main tools used for achieving OLV are the double‐lumen tube (DLT) and the bronchial blocker (BB) [1]. Despite being the gold standard for achieving OLV, DLTs are associated with complications such as a high incidence of postoperative sore throat, intubation challenges, and potential airway injury due to their large diameter [2, 3, 4, 5]. In a retrospective study, the authors reviewed their seven‐year experience using bronchial blockers (BBs) in thoracic surgery and reported that BBs effectively facilitate lung collapse in various procedures [6]. Bronchial blockers, in contrast to traditional double‐lumen endotracheal tubes, are linked to a reduced stress response and fewer postoperative airway complications [7]. Although BBs have been safely utilized to achieve OLV and provide specific advantages in certain situations, such as managing difficult airways, patients with tracheostomies, or those who are already intubated, they have not yet become the primary device of choice among anesthesiologists [8]. Surgeons frequently believe that BBs require more time to deflate the operative lung [9]. Other studies have indicated that BBs are associated with higher rates of displacement and increased costs compared to double‐lumen tubes (DLTs) [3]. However, a study conducted in 2020 demonstrated that the disconnection technique employing a BB in VATS procedures effectively reduces the time required for non‐ventilated lung collapse, achieving an overall degree of lung collapse comparable to that obtained with a DLT. This technique presents an efficient method for expediting lung collapse [10]. A study revealed that only 1.9% of participants chose BBs as their preferred device. Moreover, only 71.9% of anesthesiologists in European hospitals reported having access to these devices, suggesting that almost one‐third have limited experience with them [11]. However, the BB presents several advantages, notably its smaller diameter, which reduces the risk of damage to the patient's airway, thereby rendering it particularly suitable for pediatric patients and individuals with challenging airway anatomies. Unlike patients with a DLT who require tracheal catheter replacement when moving to the intensive care unit (ICU) post‐surgery, those with a BB can directly insert it into a single‐lumen tube for single‐lung ventilation. This approach eliminates the need for postsurgical tube replacement. However, for professionals, undergoing sufficient training to become proficient in their application can be advantageous for clinical patients.

In clinical practice, the decision to advance BB technology to facilitate lung collapse and enhance the surgical field of view presents a persistent challenge requiring resolution. During VATS, anesthesiologists frequently open the BB vent port to hasten the collapse of the non‐ventilated lung during OLV. However, our previous case observations indicate that this method may not be the most effective. In our correspondence, we documented two cases in which maintaining the vent port of the BB closed effectively promoted lung deflation, thereby achieving satisfactory surgical exposure [12]. To date, no studies have compared the efficacy of disconnection and BB techniques with vent port closure or opening methods for accelerating lung deflation during OLV in VATS. The clinical implications of these three techniques for the quality of lung collapse remain inadequately elucidated. Consequently, further randomized clinical trials are warranted to evaluate and compare the efficacy and safety of the closing vent port technique, the opening vent port technique, and the disconnection technique.

## 2 Materials and Methods

### 2.1 Research Methodology and Participant Demographics

The Ethics Committee of Zhongda Hospital, Southeast University (Approval No. 2020ZDSYLL177‐P01, 2020) approved the study, and all patients provided written informed consent. The trial was registered on November 13, 2020, prior to patient enrollment, at http://www.chictr.org/cn. The research team identified potential participants on the day preceding the scheduled surgery.

### 2.2 Inclusion and Exclusion Criteria

Individuals between the ages of 18 and 65 years from VATS were categorized as having a physical status of I or II on the basis of the American Society of Anesthesiologists (ASA) classification. After realizing the significance and objectives of the study, all patients provided voluntary written informed consent and signed the informed consent form. The exclusion criteria were patients with ASA III or higher, contraindications to bronchial blockers, prior thoracic surgery, chronic obstructive pulmonary disease or severe asthma, an FEV1 less than 80% of the predicted value, a BMI greater than 30 kg/m^2^, suspected difficult airways, consciousness or cognitive impairments, or refusal to participate. Patients whose procedures were canceled prior to the initiation of surgery were categorized as dropouts and excluded from all subsequent analyses. Conversely, patients identified with extensive pleural adhesions during surgery were retained within the modified intention‐to‐treat (mITT) population. This population included all randomized patients who underwent surgery and possessed evaluable video recordings. Individuals with extensive adhesions were excluded solely from the per‐protocol analysis. The rationale for all post‐randomization exclusions was documented in the case report form (CRF) and archived for future reference.

### 2.3 Randomization and Blinding

Patients were randomly assigned via a computer‐generated sequence into three groups: Group 1 (BB with the vent port closed), Group 2 (BB with the vent port open), and Group 3 (BB with the disconnection technique). The allocation results were concealed using sequentially numbered, sealed opaque envelopes. One investigator was responsible for assigning the numbers in sequence and sealing them within the opaque envelopes. Immediately prior to the induction of anesthesia, and following the anesthesiologist's confirmation of the absence of a potentially difficult airway, the researchers opened a sealed envelope to determine the group assignment. Complete blinding of the surgeons in Group 3 cannot be assured, as the disconnection of the breathing circuit at the moment of pleural opening may be detectable. Although the surgeons and the anesthesiologist conducting the intervention could not be blinded, the patients, video assessors, data collectors, and statisticians remained unaware of the group allocation.

### 2.4 Preoperative Preparations and Anesthesia Protocol

Preoperatively, patients did not receive any premedication. Upon entering the operating room, patients receive standard monitoring, including pulse oximetry, bispectral index (BIS) measurement, electrocardiography (ECG), venous access, and invasive blood pressure monitoring via the radial artery.

After administering oxygen and inducing anesthesia with midazolam (0.03 mg/kg), propofol (1.5 mg/kg), sufentanil (0.3 μg/kg), and rocuronium (0.6 mg/kg) for tracheal intubation, the tracheal tube cuff pressure was maintained between 25 and 30 cmH_2_O. After muscle relaxation, patients were intubated with a single‐lumen endotracheal tube (SLT) (8.0 mm internal diameter for males, 7.5 mm for females) to a depth of 22–24 cm and then secured. The Coopdech BB (Hangzhou Tappa Medical Technology Co., Hangzhou, China) was inserted via the SLT with fiberoptic bronchoscope (FOB) guidance, positioning the BB cuff in the left or right main bronchus. An experienced anesthesiologist confirmed the accurate positioning of the BB. Following the repositioning of patients to either the right or left lateral decubitus position, the BB placement was confirmed via FOB. Anesthesia was maintained using sevoflurane in oxygen and propofol, with additional administration of propofol, remifentanil, and rocuronium, guided by the BIS value and the anesthesiologist's expertise. Throughout the surgical procedure, the MAP and HR were maintained at levels no less than 20% below the baseline values. After the BB was placed, volume‐controlled two‐lung ventilation (TLV) was started. The tidal volume was adjusted to 7–8 mL/kg using ideal body weight (IBW), which was determined by the following formula: for men, IBW = 50 + 0.91 × (height in cm − 152.4), and for women, IBW = 45.5 + 0.91 × (height in cm − 152.4). The respiratory rate was set at 10–12 breaths per minute, maintaining an end‐expiratory carbon dioxide level of 35 to 45 mmHg, an I/E ratio of 1:2, and an FiO_2_ of 1.0, without using PEEP. In Groups 1 and 2, OLV was initiated by inflating the cuff with 8 mL of air once the patient was in the lateral position. Once OLV started, Group 1 immediately closed the vent port of BB, and Group 2 opened the vent port (Figure 1); then, ventilation started again. In a pilot study of 10 patients, the median surgical preparation time after lateral positioning was 12 min (range 10–14 min). Therefore, we fixed OLV to pleural incision at 13 min to standardize the lung deflation period. After 13 min of OLV before pleural incision, the surgeon is permitted to proceed with opening the pleura.

**FIGURE 1:**
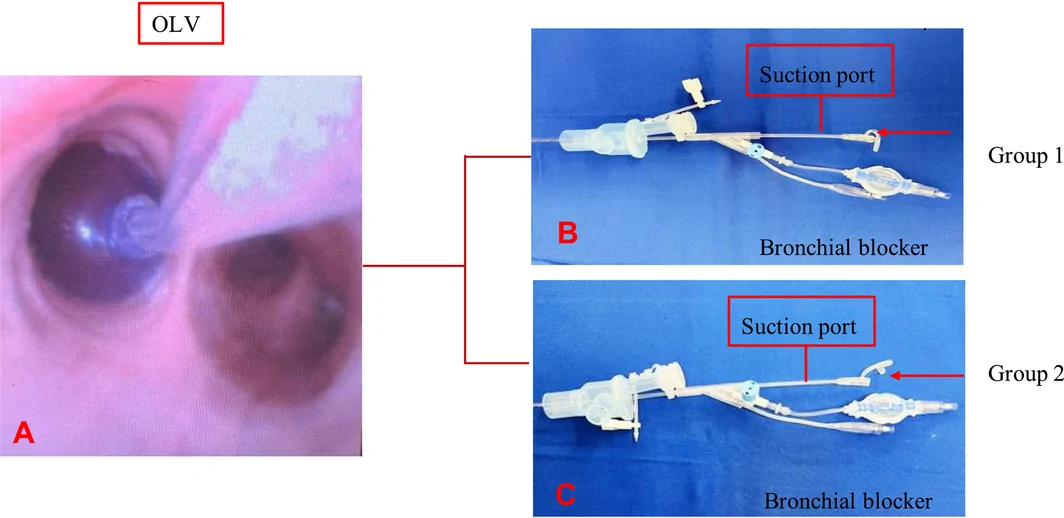
The illustration depicts the different measures taken by Group 1 and Group 2 to promote lung collapse. (A) The bronchial blocker was positioned within a primary bronchus, and the capsule was subsequently inflated to facilitate single‐lung ventilation. (B) Group 1, BB with the vent port closed; (C) Group 2, BB with the vent port open.

In conventional Group 3, upon opening the thoracic cavity, the TLV was temporarily halted for 60 s via the disconnection technique, which involved turning off the ventilator and exposing the breathing circuit to ambient air before initiating OLV, while the vent port of BB was routinely open. In accordance with protective lung ventilation guidelines, a one‐lung ventilation strategy was applied with the following parameters: tidal volume of 4–6 mL/kg (IBW), respiratory rate of 14–16 breaths/min, end‐expiratory carbon dioxide of 40–60 mmHg, I/E ratio of 1:2, FiO_2_ level of 1 [13]. Commencing with the initial skin incision, the surgeon evaluated the degree of lung collapse in the three different groups per 5 min. After surgery, patients were moved to the post‐anesthesia care unit (PACU) for monitoring and recovery.

Postoperative pulmonary collapse scores were assessed through the analysis of surgical video recordings. Following the pleural incision, video clips were captured at 5‐min intervals over a 30‐min period, specifically at 0, 5, 10, 15, 20, 25, and 30 min postincision. Due to the assessment of lung collapse at 5‐min intervals, the precise timing of complete collapse was subject to interval censoring. For the primary analysis, the event time was defined as the earliest scheduled assessment at which complete collapse was observed. Patients who did not exhibit complete collapse by the 30‐min mark were right‐censored at 30 min. Five assessors (three thoracic surgeons and two attending anesthesiologists) who took no part in the conduct of the trial independently reviewed the video clips. The clips were de‐identified: Patient identifiers, group labels, and time stamps were removed, and no clip contained a view of the airway or the breathing circuit. Clips were presented in random order across groups and time points, so that assessors were blinded both to allocation and to the time point being scored. Each assessor rated each clip on a verbal rating scale from 0 (no lung collapse) to 10 (complete collapse), taking into account the extent of collapse, the spatial arrangement of the lung within the thoracic cavity, the presence of atelectasis, and lung coloration [14]. The score for each clip was defined as the median of the five assessors' ratings. Complete lung collapse was pre‐defined as a median score of 10. The time to achieve complete collapse was defined as the earliest scheduled assessment time point at which a median score of 10 was attained and maintained at all subsequent evaluations. To ensure the accuracy of the primary outcome assessment and minimize inter‐evaluator variability, five evaluators underwent systematic training prior to the commencement of this study. The consistency among the five evaluators was quantified using two‐way random‐effects model, absolute agreement, and ICC (A, 1) within a single measurement group (ICC = 0.924 [95% CI: 0.90–0.944], indicating good consistency) (Data S2).

This study recorded the following parameters: (I) mean arterial pressure (MAP) and heart rate (HR) at T0 (before general anesthesia was induced), T1 (at the time of incision), and T2 (30 min after skin incision). Additionally, the partial pressure of oxygen (PaO_2_), partial pressure of carbon dioxide (PaCO_2_), potential of hydrogen (pH), and oxygen saturation (SaO_2_) were measured at T0 and T2. The clinical details of the patients, including age, sex, height, weight, ASA classification, surgical position, and the ratio of forced expiratory volume in 1 s to forced vital capacity (FEV1/FVC), were recorded. (II) The primary outcome was the time to complete lung collapse, which was scored according to the video clips obtained during surgery. The secondary outcome was the surgeon's satisfaction with lung collapse at surgery completion, assessed via a visual analogue scale (0 = *unsatisfied*; 10 = *very satisfied*), the incidence of hypoxemia (SpO_2_ < 90% for ≥ 15 s during OLV with FiO_2_ = 1.0, managed by a stepwise rescue protocol FOB confirmation, recruitment maneuver and positive end‐expiratory pressure [PEEP] adjustment, continuous positive airway pressure [CPAP] to the non‐ventilated lung, intermittent or continuous two‐lung ventilation after cuff deflation, and request to interrupt the procedure if needed); the duration of OLV; the total surgery duration; the frequency of bronchial blocker malpositions (FOB‐confirmed cuff malposition resulting in loss of lung isolation and requiring repositioning); and the incidence of perioperative complications (recorded from anesthesia induction to leaving the postoperative recovery unit [PACU]) included pulmonary edema, respiratory failure, or cardiovascular events during surgery. All the data were recorded by an independent anesthesiologist who was blinded to the collapse technique.

### 2.5 Sample Size and Statistical Analysis

This study is designed as a prospective, randomized trial. Sample size was estimated using PASS 15.0 for a two‐sided log‐rank test with α = 0.05 and power = 80%. Based on a pilot study (*n* = 10), the 30‐min event rate (complete collapse) in the control group (open vent port) was approximately 40%. A hazard ratio (HR) of 2.0 was considered clinically meaningful, corresponding to a reduction in median collapse time from 25.2 min to approximately 12.6 min. Under these assumptions, the required sample size was 23 patients per group. Accounting for a 10% dropout rate, we enrolled 25 patients per group (total 75).

Data analysis was conducted using R (version 4.5.1). Continuous variables were tested for normality using the Shapiro–Wilk test. Normally distributed data were presented as mean ± SD and were compared among the three groups using one‐way ANOVA with Bonferroni correction. Non‐normally distributed data were presented as median (IQR) and were compared using the Kruskal–Wallis test with Dunn's post hoc test and Bonferroni correction. For the primary outcome (time to complete lung collapse), Kaplan–Meier curves were generated, and the log‐rank test was used for pairwise comparisons with Bonferroni adjustment. Patients who did not achieve complete collapse within the 30‐min observation period were right‐censored at 30 min, consistent with standard survival analysis for a fixed follow‐up time. This approach assumes that collapse could have occurred after 30 min, and the hazard ratio should be interpreted with this in mind. Cox proportional hazards regression was performed to estimate hazard ratios (HRs) with 95% confidence intervals (CIs). The proportional hazards assumption was verified using scaled Schoenfeld residuals. Due to the violation of the proportional‐hazards assumption, as indicated by a global *p* value of 0.038, we conducted a restricted mean survival time (RMST) analysis over a 30‐min period. It is important to note that this RMST analysis was not prespecified and should be considered exploratory or supplementary in nature. The purpose of this analysis was to offer a clinically interpretable effect measure within the fixed 30‐min observation period.

For repeated measurements (MAP and HR at T0, T1, T2), a linear mixed‐effects model was used with group, time, and group‐by‐time interaction as fixed effects and patient as random intercept. Post hoc pairwise comparisons were adjusted using Bonferroni correction. For blood gas parameters measured at two time points (T0, T2), a two‐way repeated‐measures ANOVA (group × time) was performed. Categorical variables were compared using Fisher's exact test. Surgeon satisfaction scores were compared using the Kruskal–Wallis test with post hoc Dunn's test (Bonferroni correction). A two‐sided *p* < 0.05 was considered statistically significant.

## 3 Results

### 3.1 Study Population

The flowchart for the study is depicted in Figure 2. There were 112 patients screened, and 37 were not enrolled: 22 declined participation, 12 did not meet inclusion criteria, and 3 were excluded for other reasons. Consequently, 75 patients were randomly assigned to three groups: Group 1, Group 2, or Group 3. Three patients were excluded due to failed video recording, and 72 patients were included in the final mITT analysis, which was the primary analysis of this study. A per‐protocol analysis (excluding 5 patients with pleural adhesions, *n* = 67) was performed as a sensitivity analysis (Data S1).

**FIGURE 2:**
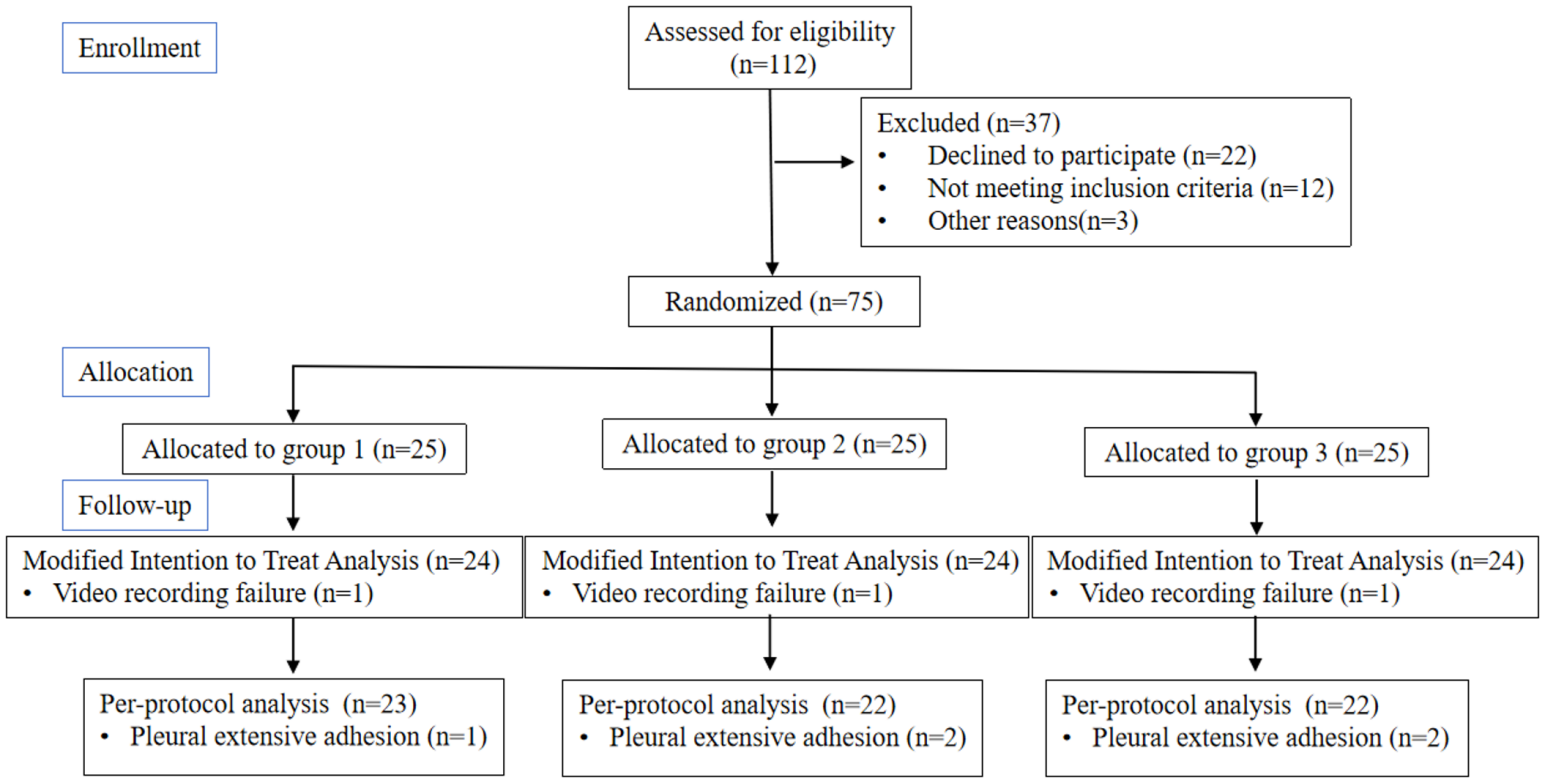
A CONSORT flow chart illustrating the study design and participants' progress. CONSORT: Consolidated Standards of Reporting Trials. Group 1, BB with the vent port closed; Group 2, BB with the vent port open; Group 3, the disconnection technique.

The three groups did not show any significant differences in patient demographics, surgical position, or lung function (*p* > 0.05) (Table 1).

**TABLE 1:** Patient demographic characteristics.

Variable	Group 1 (*n* = 24)	Group 2 (*n* = 24)	Group 3 (*n* = 24)	*p*
Age (years)	56.7 ± 11.3	56.1 ± 13.9	63.4 ± 12.2	0.089
Sex, *n* (M/F)	8/16	11/13	13/11	0.385
Height (cm)	164.2 ± 7.6	163.3 ± 6.98	164.4 ± 7.7	0.846
Weight (kg)	65.8 ± 11.2	62.5 ± 8.5	64.9 ± 10.8	0.515
BMI (kg/m^2^)	24.3 ± 2.8	23.7 ± 3.4	24.9 ± 3.4	0.806
ASA (I/II)	9/15	10/14	7/17	0.748
Surgical position, *n* (L/R)	13/11	11/13	13/11	0.873
FEV1/FVC (%)	77.4 ± 4.8	77.8 ± 5.4	75.3 ± 7.4	0.298
Type of surgery, *n*				0.797
Lobectomy	12	12	9	
Segmentectomy	7	5	7	
Wedge resection	5	7	8	

*Note:* Data are presented as the mean ± SD or counts.

Abbreviations: ASA, American Society of Anesthesiologists; BMI, body mass index; F, female; FEV1, forced expiratory volume in 1 s; FVC, forced vital capacity; L, left; M, male; R, right.

### 3.2 Primary Outcome: Time to Complete Lung Collapse

The observation period was fixed at 30 min after pleural incision. During follow‐up, 54 patients (75%) reached the endpoint; the remaining 18 patients were right‐censored because they did not achieve complete collapse within 30 min. Kaplan–Meier analysis revealed a significant overall difference among the three groups in the time to complete lung collapse (log‐rank χ^2^ = 37.153, df = 2, *p* < 0.001; Table 2). Pairwise comparisons using the log‐rank test with Bonferroni correction for multiple testing showed no significant difference between Group 1 and Group 3 (χ^2^ = 0.439 *p* = 0.508; adjusted *p* = 1.000). In contrast, both Group 1 and Group 3 differed significantly from Group 2 (Group 1 vs. Group 2: χ^2^ = 33.151, adjusted *p* < 0.001; Group 2 vs. Group 3: χ^2^ = 27.947, adjusted *p* < 0.001).

**TABLE 2:** Log‐rank tests for time to complete lung collapse.

Comparison	χ^2^	df	*p*	Bonferroni‐adjusted *p*
Overall (three groups)	37.153	2	<0.001	*—*
Group 1 vs. Group 2	33.151	1	< 0.001	< 0.001
Group 1 vs. Group 3	0.439	1	0.508	1.000
Group 2 vs. Group 3	27.947	1	< 0.001	< 0.001

*Note:* Complete lung collapse was defined as a collapse score of 10 within a 30‐min observation period after pleural incision. The overall log‐rank test compared the three groups simultaneously. Pairwise comparisons were performed using the log‐rank test, and *p* values were adjusted for three comparisons by the Bonferroni method. A Bonferroni‐adjusted *p* value < 0.05 was considered statistically significant. df, degrees of freedom. —, not applicable.

In Figure 3, the median time to complete collapse for Group 1 and Group 3 was 15 min (95% CI: 10–15 min for both). For Group 2, the median could not be estimated because the survival probability remained above 0.5 throughout the observation period; the lower bound of the median was 30 min. At 30 min, the cumulative incidence of complete collapse was 95.8% (23/24) of patients in Group 1, 91.7% (22/24) in Group 3, but only 37.5% (9/24) in Group 2.

**FIGURE 3:**
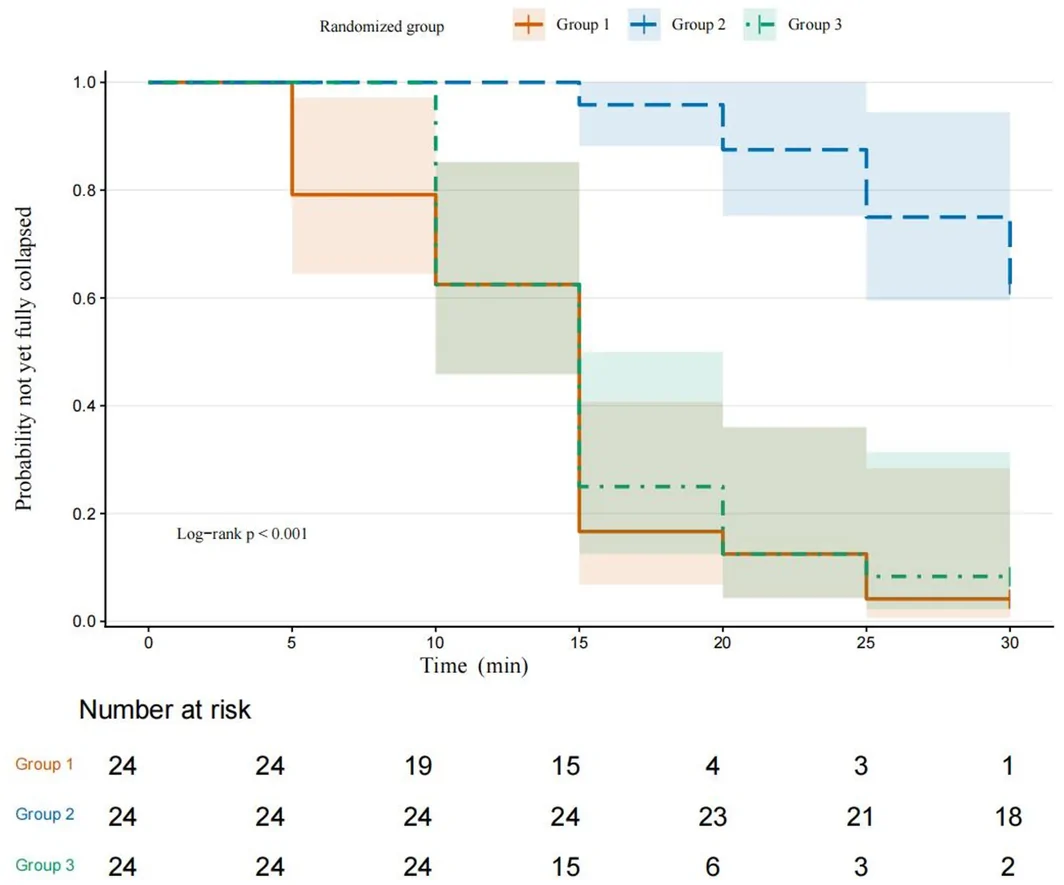
Kaplan–Meier curves for time from pleural opening to complete lung collapse in the mITT population. The y‐axis shows the probability of not yet achieving complete lung collapse; shaded areas are 95% confidence intervals, censor marks indicate participants not fully collapsed at 30 min, and the risk table gives the number remaining under observation. Overall log‐rank χ^2^ = 37.153 (df = 2), *p* < 0.001. Group 1, BB with the vent port closed; Group 2, BB with the vent port open; Group 3, BB with the disconnection technique.

In Table 3, Cox proportional hazards regression was performed to quantify the relative speed of collapse. Group 1 vs. Group 2 (HR = 9.80, 95% CI: 4.22–22.73, *p* < 0.001), Group 3 vs. Group 2 (HR = 7.630, 95% CI: 3.36–17.31, *p* < 0.001), and Group 1 vs. Group 3 (HR = 1.20 [0.67–2.16], *p* = 0.538). Taken together, the time to complete lung collapse was markedly longer in Group 2 than in Group 1 or Group 3, whereas there was no statistical difference between Group 1 and Group 3. The primary analysis employed the log‐rank test, in alignment with the sample size calculation. Due to the violation of the proportional hazards assumption (global *p* = 0.038), Cox hazard ratios are presented as secondary time‐averaged estimates. In an exploratory analysis using the 30‐min RMST, complete collapse occurred, on average, 14.17 min earlier in Group 1 (95% CI: 11.10–17.23) and 12.50 min earlier in Group 3 (95% CI: 9.62–15.38) compared to Group 2. No significant difference was observed between Groups 1 and 3 (1.67 min, 95% CI: −1.85 to 5.19) (Table 4).

**TABLE 3:** Cox proportional hazards regression for time to complete lung collapse.

(Reference: Group 1; secondary analysis)		
Comparison	HR (95% CI)	*p*
Group 2 vs. Group 1	0.102 (0.044–0.237)	< 0.001
Group 3 vs. Group 1	0.832 (0.463–1.494)	0.538

(Reference: Group 2)		
Comparison	HR (95% CI)	*p*
Group 1 vs. Group 2	9.80 (4.22–22.73)	< 0.001
Group 3 vs. Group 2	7.630 (3.364–17.308)	< 0.001

(Reference: Group 3)		
Comparison	HR (95% CI)	*p*
Group 1 vs. Group 3	1.20 (0.67–2.16)	0.538
Group 2 vs. Group 3	0.131 (0.058–0.297)	< 0.001

*Note:* The proportional‐hazards assumption was not met (global Schoenfeld, *p* = 0.038); therefore, Cox HRs are secondary time‐averaged estimates. The 30‐min RMST analysis was exploratory and not prespecified. The 30‐min RMST was 13.75, 27.92, and 15.42 min in Groups 1, 2, and 3, respectively. Group 2 exceeded Group 1 by 14.17 min (95% CI: 11.10–17.23) and Group 3 by 12.50 min (95% CI: 9.62–15.38); there was no significant difference between Groups 1 and 3, as indicated by a mean difference of 1.67 min (95% CI: −1.85 to 5.19).

Abbreviations: CI, confidence interval; HR, hazard ratio.

**TABLE 4:** Survival summary for complete lung collapse (mITT).

Group	*n*	Events/censored	Median time, min (95% CI)	Not fully collapsed at 30 min	30‐min RMST, min	RMST SE
Group 1	24	23/1	15 (10–15)	1 (4.2%)	13.75	1.33
Group 2	24	9/15	NR (30–NR)	15 (62.5%)	27.92	0.83
Group 3	24	22/2	15 (10–15)	2 (8.3%)	15.42	1.21

*Note:* The 30‐min RMST was 13.75, 27.92, and 15.42 min, respectively. Group 2 exceeded Group 1 by 14.17 min (95% CI: 11.10–17.23) and Group 3 by 12.50 min (95% CI: 9.62–15.38); Groups 1 and 3 exhibited no significant difference (mean difference of 1.67 min, 95% confidence interval: −1.85 to 5.19).

### 3.3 Secondary Outcomes

There was no significant difference in OLV duration or surgical time across the three groups. Intraoperative hypoxemia occurred in one patient from Group 1, and one patient from each group experienced blocker malposition. This patient was managed by the FOB confirmation, augmentation of FiO_2_ and PEEP adjustments; no escalation to recruitment maneuver, CPAP, or two‐lung ventilation was required.

No patients in any of the groups experienced perioperative complications, and there was no significant difference between the groups in the duration of one‐lung ventilation or in total surgical time (see Table 5). Blood analysis and hemodynamic data collected 30 min after the initiation of surgery under general anesthesia are presented in Tables 5 and 6. Compared with those at baseline (T0), at T2, intragroup comparisons revealed increases in PaO_2_, PaCO_2_, and SaO_2_, while pH decreased (*p* < 0.001). The groups presented comparable values for PaO_2_, PaCO_2_, SaO_2_, and pH, with no notable differences (*p* > 0.05) (see Table 6).

**TABLE 5:** Outcomes of the research.

Variables	Group 1 (*n* = 24)	Group 2 (*n* = 24)	Group 3 (*n* = 24)	*p*
Median time to complete lung collapse (min)	15 (95% CI: 10–15)	NR (lower bound: > 30)^c^	15 (95% CI: 10–15)	< 0.001^a^
Cumulative incidence of complete collapse at 30 min, *n* (%)	23 (95.8)	9 (37.5)	22 (91.7)	< 0.001^b^
Intraoperative hypoxemia, *n* (%, 95% CI)	1 (4.2, 0.1–21.1)	0 (0, 0–14.3)	0 (0, 0–14.3)	> 0.999
Number of bronchial blocker malpositions, *n* (%, 95% CI)	1 (4.2, 0.1–21.1)	1 (4.2, 0.1–21.1)	1 (4.2, 0.1–21.1)	> 0.999
Perioperative complications, *n* (%, 95% CI)	0 (0, 0–14.3)	0 (0, 0–14.3)	0 (0, 0–14.3)	1.000
Duration of surgery (min)	147.8 ± 57.41	145.8 ± 79.57	127.5 ± 47.48	0.471
Duration of OLV (min)	122.2 ± 58.91	118.8 ± 60.78	96.0 ± 45.05	0.212

*Note:* Continuous data are mean ± SD and median (95% CI), or *n* (%), as appropriate. Categorical data are presented as *n* (%, 95% CI). Exact 95% CI were calculated using the Clopper–Pearson method.

Abbreviations: NR, median not reached within the 30‐min observation window; OLV, one‐lung ventilation.

^a^ Overall log‐rank test.

^b^ Pearson chi‐square test for complete lung collapse by 30 min.

^c^ The median time could not be estimated because the survival probability remained above 0.5 throughout the 30‐min observation period; the lower bound of the median was 30 min.

**TABLE 6:** Arterial blood gas analysis.

Variables	Group	T0	T2
PaO_2_ (mmHg)	Group 1	87.6 ± 7.8	266.6 ± 109.4*
	Group 2	85.6 ± 6.1	275.5 ± 121.9*
	Group 3	83.4 ± 8.1	225.6 ± 95.6*
PaCO_2_ (mmHg)	Group 1	35.0 ± 3.5	46.5 ± 8.5*
	Group 2	35.0 ± 4.1	43.3 ± 9.1*
	Group 3	35.1 ± 3.9	45.2 ± 9.9*
pH	Group 1	7.420 ± 0.028	7.342 ± 0.060*
	Group 2	7.423 ± 0.026	7.391 ± 0.079
	Group 3	7.408 ± 0.033	7.353 ± 0.067*
SaO_2_ (%)	Group 1	96.87 ± 0.91	99.58 ± 0.52*
	Group 2	97.16 ± 1.16	99.34 ± 0.91*
	Group 3	96.78 ± 1.63	98.72 ± 1.84*

*Note:* Data are mean ± SD.

Abbreviations: PaCO_2_, arterial partial pressure of carbon dioxide; PaO_2_, arterial partial pressure of oxygen; pH, potential of hydrogen; SaO_2_, arterial oxygen saturation.

* Holm‐adjusted *p* < 0.05 versus T0.

Table 7 indicates that there were no statistically significant differences in MAP and HR between the groups. Intragroup comparisons revealed a decrease in the MAP at T0 and T1 in Groups 1 and 3, whereas Group 2 showed a decline in the MAP at T1 compared with T0 (*p* < 0.05). Surgeon satisfaction scores were significantly higher in Group 1 (median 8, IQR 7–8) and Group 3 (median 9, IQR 7–9) than in Group 2 (median 4, IQR 4–5; *p* < 0.001 for both comparisons). No significant difference was observed between Group 1 and Group 3 (*p* = 0.75) (Figure 4).

**TABLE 7:** Hemodynamic analysis.

Variables	Group	T0	T1	T2
MAP (mmHg)	Group 1	100.3 ± 17.5	94.7 ± 13.8	80.9 ± 9.8*
	Group 2	93.5 ± 13.9	91.1 ± 18.5	80.9 ± 12.8*
	Group 3	101.5 ± 13.3	95.9 ± 12.4	76.2 ± 9.8*
HR (bpm)	Group 1	73.8 ± 12.7	66.5 ± 11.6	70.0 ± 12.5
	Group 2	72.3 ± 12.3	66.5 ± 12.5	66.8 ± 8.9
	Group 3	72.2 ± 8.3	68.8 ± 10.8	66.8 ± 8.3

*Note:* Data are mean ± SD. Group × time interaction: MAP *p* = 0.128; HR *p* = 0.602.

Abbreviations: HR, heart rate; MAP, mean arterial pressure.

* Holm‐adjusted *p* < 0.05 versus T0.

**FIGURE 4:**
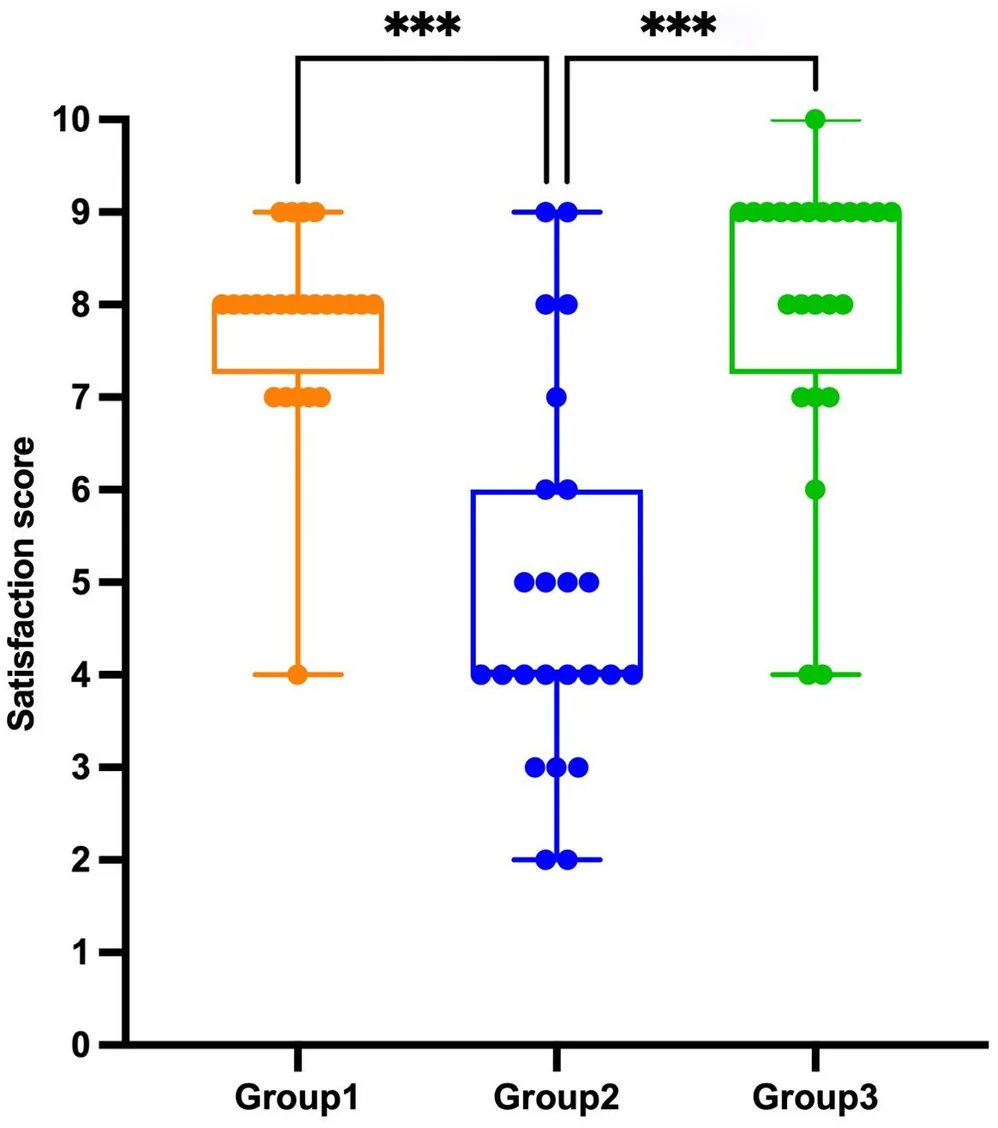
The surgeon satisfaction scores across the three groups are depicted, with boxes indicating the interquartile range (IQR), horizontal lines representing the medians, and whiskers extending to the most extreme values within 1.5 times the IQR. Individual data points are superimposed. Comparative analyses between groups were conducted using the Kruskal–Wallis test, followed by Dunn's post hoc test with Bonferroni correction. Statistical significance is denoted as ***p < 0.001. Group 1, BB with the vent port closed; Group 2, BB with the vent port open; Group 3, BB with the disconnection technique.

## 4 Discussion

A comparison of the effects of three different techniques on the collapse of the non‐ventilated lung during OLV was conducted in this randomized controlled trial. The time to complete lung collapse was significantly shorter in both Group 1 and Group 3 compared to Group 2, with no significant difference between Group 1 and Group 3. The surgeon's satisfaction score with the operating space was significantly higher in Group 1 and Group 3 than in Group 2. No statistically significant difference in satisfaction scores was observed between Group 1 and Group 3. Although the total operation time was not shortened, the better surgical field reduces the clamping and traction of the lung tissue by the operator, which may have potential protective significance from the perspective of reducing lung mechanical injury and postoperative inflammatory reaction. In Group 3, the application of the disconnection technique necessitated the disconnection of both the ventilator and the patient's tracheal catheter. This process, when inhalation anesthesia is employed, results in the release of an aesthetic gases into the operating‐room environment; this potential exposure was not measured in the present study and is raised only as a hypothesis. The time to complete lung collapse was significantly shorter in both Group 1 and Group 3 compared with Group 2. Although no statistically significant difference was found between Group 1 and Group 3, this finding should not be interpreted as evidence of equivalence or that one technique can replace the other. Our study was not designed as an equivalence or non‐inferiority trial.

Within the first 30 min of the surgical video clips, significant variations were observed in the distribution of complete lung collapse among the three groups. The median time required for lung collapse was 15 min for both Group 1 and Group 3, whereas it could not be estimated, with the lower bound of the median being 30 min for Group 2. Furthermore, the occurrence of total lung collapse within 30 min following pleural opening was observed to be 95.8% in Group 1 and 91.7% in Group 3, whereas it was significantly lower at 37.5% in Group 2 (*p* < 0.001). Compared with those at T0, intragroup analyses at T2 demonstrated an increase in PaO_2_, PaCO_2_, and SaO_2_, accompanied by a decrease in pH. This alteration is likely attributable to the transition from TLV to OLV, which may have induced a ventilation–perfusion imbalance associated with OLV.

The process of lung collapse can be delineated into two distinct phases, each characterized by unique mechanisms and contributions to overall collapse [15]. The initial stage of pulmonary collapse is initiated immediately upon exposure of the nonventilated lung to ambient air, driven by the intrinsic elastic recoil properties of the lung tissue, and persists until the sealing of minor airways occurs. Alternatively, the following phase is chiefly determined by the diffusion of gases and the phenomenon of absorption atelectasis [16]. Various methods have been established to support lung collapse in the context of thoracoscopic surgery, including the disconnection technique, carbon dioxide insufflation, bronchial suction, and inhalation of N_2_O or 100% O_2_ [17, 18, 19, 20]. During OLV prior to pleural opening, if the lumen of the lung isolation device remains open, environmental air can be drawn into the nonventilated lung. Our group previously noted that during OLV before pleural incision, a U‐shaped tube containing a combination of 1 mL of methylene blue and 1 mL saline was linked to the lung that was not ventilated via the vent port of a BB. We subsequently identified substantial negative pressure, which caused air to move in Figure 5 (Video S1), which was generally consistent with findings from previous studies [15, 21]. It is crucial to acknowledge that the two efficacious techniques may have functioned through distinct mechanisms. In Group 1, the closure of the vent port likely inhibited the entry of ambient air into the non‐ventilated lung prior to pleural opening, thereby preserving its intrinsic elastic recoil and facilitating unimpeded absorption atelectasis. Conversely, in Group 3, the vent port remained open before pleural opening; thus, the observed benefit was likely attributable primarily to the disconnection maneuver at the moment of pleural opening, which permitted the rapid egress of gas from the non‐ventilated lung. Given that the trial was not designed to differentiate between pre‐pleural and post‐pleural effects, we are unable to ascertain the relative contributions of each mechanism. This distinction should be regarded as hypothesis‐generating and merits further investigation. In contrast, the prolonged duration of lung deflation in Group 2 (BB with the vent port open) was associated with the movement of air into the nonventilated lumen, a result of mediastinal shifts occurring during OLV prior to pleural opening. The delay is caused by the passive entry of surrounding air into the nondependent lung, leading to a substantial replacement of oxygen with nitrogen. The slow diffusion of nitrogen into the lungs significantly prevents it from collapsing [22, 23].

**FIGURE 5:**
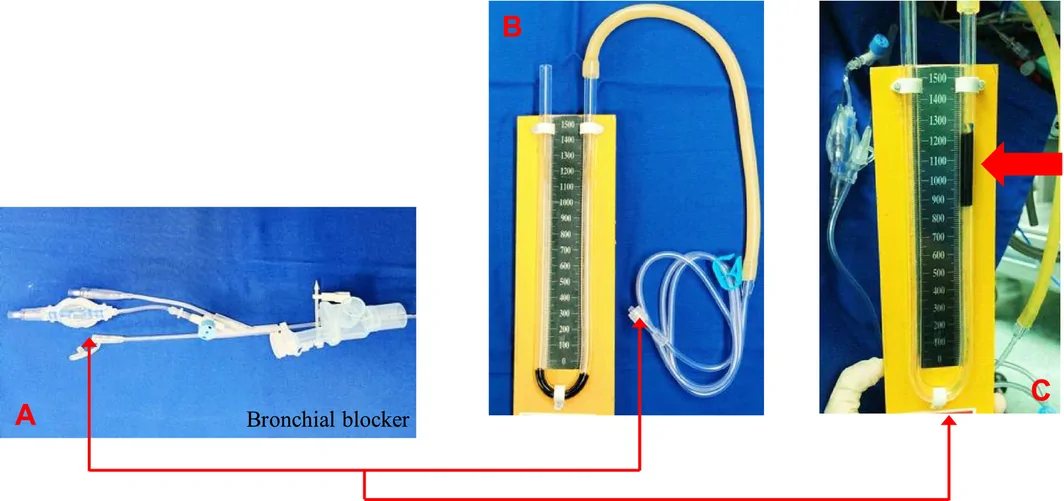
A notable negative pressure was observed, accompanied by the influx of air during OLV utilizing a BB. In (A), the vent port of a BB was linked to the U‐shaped tube connector depicted in (B). Prior to pleurotomy, during unilateral lung ventilation, the methylene blue solution within the U‐shaped tube, as illustrated in (C), was drawn into the lung on the non‐ventilated side.

Zhang et al. demonstrated that commencing OLV at least 6 min before incision into the pleural cavity, thereby establishing a restricted environment for the lung without ventilation while breathing pure oxygen, can facilitate more rapid lung collapse during VATS [24]. Another study demonstrated that maintaining the bronchoscopy access to the surgical lung is open for an approximate time span of 12 min before the pleural cavity incision allows a substantial amount of air from the environment to flow into the lung, thereby decelerating the rate of lung collapse [15]. Drawing on the aforementioned studies, this research established that the duration from one‐lung ventilation (OLV) to pleural opening was 13 min for both Groups 1 and 2. We posit that a 13‐min interval is adequate to observe the dynamics of gas exchange and, consequently, to examine the collapse mass effectively. In addition, after the patient is positioned laterally, OLV is initiated. The surgeon subsequently proceeds with handwashing and skin sterilization.

The study was subject to numerous limitations. First, the approach to assessing lung collapse via a rating scale was inherently subjective, necessitating the development of objective alternatives in future research. Second, the sample size calculation assumed a hazard ratio of 2.0, whereas the observed effect was much larger; this reflects the limited precision of the pilot study but does not invalidate the findings. Third, the study participants ranged in age from 18 to 65 years, were classified as ASA I or II, and exhibited normal pulmonary function prior to undergoing their surgical procedures. Finally, the trial is unable to separate the effects of pre‐pleural air entry prevention from post‐pleural gas efflux, as the interventions differed in timing. Given that the participants in the study were aged 18–65 years and classified with an ASA physical status of I–II, alongside normal spirometry results, the findings should not be generalized to elderly patients (aged over 65 years) or those with chronic obstructive pulmonary disease, air trapping, limited oxygen reserve, difficult airways, or an ASA status of III–IV. Consequently, the efficacy of this technique for inducing lung collapse in elderly patients (aged over 65 years) with compromised pulmonary function or those classified as ASA grade III or higher remains uncertain. Additional multicenter randomized clinical trials are crucial for investigating and assessing the methodologies utilized to achieve lung collapse, with the aim of improving the effectiveness of thoracoscopic surgery.

## 5 Conclusion

In patients aged 18–65 years with ASA I‐II and normal pulmonary function undergoing thoracic surgery, the closure of the BB vent port results in a more rapid lung collapse compared to maintaining the vent port open. However, there was no statistically significant difference observed between the vent port closure technique and the disconnection technique regarding the time required for complete lung collapse.

## Author Contributions

**Juan Yao** and **Jie Sun:** study design, comprehensive revision of the manuscript. **Juan Yao:** prepare the manuscript. **Fang‐Ming Shen** and **Jiao‐Jiao Yang:** patient recruitment and data collection. **Jue Xie** and **Lu‐Xin Huang:** data analysis. **Xiao‐Xiang Tan** and **Ya‐Jing Chen:** participate in the assessment of lung collapse. **Ying Yang** and **Jie Sun:** edited the manuscript. The manuscript was reviewed by all authors.

## Funding

This study was supported by the Zhongda Hospital Affiliated to Southeast University, Jiangsu Province High‐Level Hospital Construction Funds (Grant No. YKK24267).

## Ethics Statement

The Ethics Committee of Zhong Da Hospital, Southeast University (Approval No. 2020ZDSYLL177‐P01, 2020) approved the study, and all patients provided written informed consent.

## Conflicts of Interest

The authors declare no conflicts of interest.

## Supporting information


**Data S1:** Supporting information.

**Data S2:** Supporting information.

**Video S1:** Supporting information.

## Data Availability

The deidentified dataset supporting the conclusions of this study will be made available upon reasonable request to the corresponding author.
