# Supplementary material for: Closed Versus Open Vent Port Techniques and the Disconnection Technique for Optimizing Lung Collapse With Bronchial Blockers in Video‐Assisted Thoracoscopic Surgery: A Randomized Controlled Trial
Source: Clin Respir J. 2026 Oct 22;20(10):e70235. doi: 10.1111/crj.70235 (PMC13691421; doi:10.1111/crj.70235)
Supplement: Supplementary file 1 [file CRJ-20-e70235-s002.docx]

**SPSSAU - ICC Intraclass Correlation Coefficient**

| ICC Intraclass Correlation Coefficient Results | | |
| --- | --- | --- |
| Two-Way Mixed/Random \| ICC Value \| 95% CI \| \| Absolute Agreement | ICC Value | 95% CI |
| Single Measure ICC(A,1) | 0.924 | 0.900 – 0.944 |
| Average Measure ICC(A,K) | 0.984 | 0.978 – 0.988 |
| Note: A = absolute agreement, 1 = single measure, K = average measure | | |
