# Supplementary material for: Closed Versus Open Vent Port Techniques and the Disconnection Technique for Optimizing Lung Collapse With Bronchial Blockers in Video‐Assisted Thoracoscopic Surgery: A Randomized Controlled Trial
Source: Clin Respir J. 2026 Oct 22;20(10):e70235. doi: 10.1111/crj.70235 (PMC13691421; doi:10.1111/crj.70235)
Supplement: Supplementary file 2 [file CRJ-20-e70235-s003.docx]

Table 2.Log-rank tests for time to complete lung collapse

| **Comparison** | **χ²** | **df** | ***p* value** | ***Bonferroni‑adjusted p*** |
| --- | --- | --- | --- | --- |
| Overall (three groups) | 47.5 | 2 | <0.0001 | *—* |
| Group 1 vs. Group 2 | 37.06 | 1 | 1.146×10⁻⁹ | *<0.001* |
| Group 1 vs. Group 3 | 0.05 | 1 | 0.823 | *1.000* |
| Group 2 vs. Group 3 | 38.30 | 1 | 6.061×10⁻¹⁰ | *<0.001* |

Complete lung collapse was defined as a collapse score of 10 within a 30‑min observation period after pleural incision. The overall log‑rank test compared the three groups simultaneously. Pairwise comparisons were performed using the log‑rank test, and *p* values were adjusted for three comparisons by the Bonferroni method. A Bonferroni‑adjusted *p* value < 0.05 was considered statistically significant. df, degrees of freedom. —, not applicable.

Table 3. Cox proportional hazards regression for time to complete lung collapse .

| **Comparison** | **HR (95% CI)** | ***P value*** |
| --- | --- | --- |
| Group 1 vs. Group 2 | 16.55 (6.29–43.59) | *<0.0001* |
| Group 3 vs. Group 2 | 15.99 (5.96–42.90) | < 0.0001 |
| Group 1 vs. Group 3 | 1.036 (0.576 – 1.863) | 0.907 |

HR, hazard ratio; CI, confidence interval. A HR < 1 indicates a lower risk (slower collapse) compared with the reference group. The proportional hazards assumption was verified by testing the scaled Schoenfeld residuals (global P = 0.48). Group 1, BB with the vent port closed; Group 2, BB with the vent port open; Group 3, the disconnection technique.

Figure 3: Kaplan‑Meier curves for time to complete lung collapse within 30 minutes after pleural incision.The vertical axis represents the probability of not reaching complete collapse (score = 10). The horizontal axis shows time in minutes. Group 1 (red), Group 2 (blue), and Group 3 (green). The number of patients at risk at each time point is shown below the graph. The log‑rank test indicated a significant overall difference among the three groups (Overall log-rank χ² = 47.5(df = 2), *p* < 0.001). Group 1(red), BB with the vent port closed; Group 2(blue), BB with the vent port open; Group 3(green) , BB with the disconnection technique.
